# Latent profiles of basic psychological needs and their associations with health risk behaviors among engineering college students

**DOI:** 10.3389/fpubh.2026.1809028

**Published:** 2026-04-22

**Authors:** Haitao Niu, Bochuan Zhao

**Affiliations:** 1 School of Physical Education, Shandong University, Jinan, China; 2 School of Physical Education and Health, Guilin University, Guilin, Guangxi, China

**Keywords:** basic psychological needs, engineering college students, health risk behaviors, latent profile analysis, self-determination theory

## Abstract

**Objective:**

Grounded in self-determination theory (SDT), this study aimed to identify latent profiles of basic psychological needs (autonomy, competence, and relatedness) among engineering college students and to examine between-profile differences in health risk behaviors.

**Methods:**

Using convenience sampling, 251 undergraduate engineering students at a university in Shandong Province, China, were surveyed between May and June 2021. Measures included basic psychological need satisfaction and seven health risk behavior indicators (unhealthy weight control, traffic risk behavior, bullying victimization, loneliness, suicide risk, smoking, and internet addiction). Latent profile analysis (LPA) was employed to identify distinct need satisfaction patterns. Multivariate analysis of variance (MANOVA) and one-way analysis of variance (ANOVA) were conducted to compare health risk behaviors across profiles.

**Results:**

Three latent profiles were identified: a low-need-satisfaction profile (74.5%), a moderate-need-satisfaction profile (19.9%), and a high-need-satisfaction profile (5.6%). MANOVA revealed a significant overall difference in health risk behaviors across profiles (Pillai’s Trace = 0.103, *F* = 1.88, *p* = 0.026). Specifically, significant between-profile differences were found for suicide risk (*F* = 3.14, *p* = 0.045, *η*^2^ = 0.025) and smoking (*F* = 6.03, *p* = 0.003, *η*^2^ = 0.046). *Post hoc* comparisons indicated that students in the low-need-satisfaction profile reported significantly higher suicide risk than those in the moderate-need-satisfaction profile (*p* = 0.033), whereas students in the high-need-satisfaction profile reported significantly more frequent smoking than those in the low-need-satisfaction profile (*p* = 0.013).

**Conclusion:**

Basic psychological need satisfaction among engineering college students is heterogeneous, and students with low need satisfaction were associated with elevated suicide risk. These findings support SDT’s predictions regarding the association between need satisfaction and psychological adjustment, and provide preliminary evidence that may inform targeted mental health interventions in higher education.

## 1 Introduction

College student mental health is a major global public health concern, and health risk behaviors—as key determinants of psychological well-being—have attracted growing attention (1). Engineering students face unique mental health challenges attributable to heavy academic workloads, rigorous curricula, and relatively limited social resources (2). Existing evidence indicates that suicidal ideation, smoking, internet addiction, and bullying victimization are prevalent among college students and exert far-reaching effects on physical and mental health (3). Nevertheless, most research has focused on predictors of single risk behaviors, with few studies adopting an integrative perspective that examines the systematic associations between individuals’ internal psychological resources and multiple risk behaviors.

Self-determination theory (SDT) provides a well-established theoretical framework for understanding psychological functioning and behavioral adaptation (4). Central to SDT is the concept of basic psychological needs (BPN)—namely autonomy, competence, and relatedness—which are considered essential prerequisites for psychological health and adaptive behavior (5). A substantial body of research has demonstrated that need satisfaction is positively associated with favorable psychological outcomes, whereas need frustration is linked to maladaptive functioning and problem behaviors (6). Among college students, BPN satisfaction is inversely related to depressive and anxiety symptoms (7) and positively associated with life satisfaction and subjective well-being (8).

In recent years, the application of SDT to health risk behavior research has expanded considerably. Ng et al. (9) reported that greater BPN satisfaction was associated with healthier dietary behaviors. Autonomy frustration has been significantly linked to suicidal ideation, and need satisfaction may serve a protective function by enhancing individuals’ sense of meaning and belonging (10). In the domain of internet addiction, BPN frustration is regarded as a key driver of excessive internet use, as individuals seek compensatory gratification online (11). Furthermore, deficits in relatedness are closely associated with bullying victimization, as insufficient social connectedness renders individuals more vulnerable to peer aggression (12).

Research has documented that engineering programs are characterized by heavy course loads, highly standardized curricula with limited elective flexibility, and a cultural ethos of “making it through” adversity, which are conditions that may systematically constrain students’ sense of autonomy over their academic choices (13, 14). Competence needs may be further threatened by the competitive and evaluative culture prevalent in engineering programs, where frequent academic failure experiences are normalized (15). The relative gender homogeneity of engineering programs, where female students remain significantly underrepresented (16), may additionally limit the richness and diversity of peer social networks, creating structural barriers to relatedness satisfaction. According to BPNT, when basic psychological needs are chronically frustrated, individuals are prone to dysregulated coping and compensatory behaviors aimed at substituting for unmet needs (5), which may manifest as engagement in health risk behaviors such as suicidal ideation, smoking, or internet addiction. Taken together, the distinctive educational environment of engineering students provides strong *a priori* justification for expecting lower and more varied BPN satisfaction compared with students in other disciplines, and for anticipating that BPN frustration will manifest in elevated health risk behaviors in this population. Yet the extent to which engineering students differ in their BPN profiles, and how such profiles relate to specific risk behaviors, remains unclear, underscoring the need for a person-centered analytic approach.

However, traditional variable-centered approaches assume sample homogeneity and are unable to capture individual differences and group heterogeneity in BPN satisfaction patterns. In practice, individuals may exhibit different configurations of satisfaction across the three needs. For instance, some individuals may experience adequate autonomy and competence satisfaction but relatively low relatedness, while others may have uniformly low satisfaction across all three needs. Latent profile analysis (LPA), a person-centered analytic method, can identify latent subgroups sharing similar psychological characteristics and reveal within-population heterogeneity (17). A limited number of studies have employed LPA to examine BPN profiles. Gu and Wang (8) identified multiple need satisfaction profiles among college students—including “high satisfaction,” “low satisfaction,” and “moderate satisfaction” types—and found that these profiles differed significantly in psychological adjustment outcomes. Ratelle et al. (18), drawing on SDT, identified heterogeneous academic motivation profiles among college students characterized by varying combinations of autonomous and controlled motivation (e.g., autonomous, high-autonomous–high-controlled), and demonstrated that these motivation profiles were significantly associated with academic performance and persistence. However, no study to date has combined BPN profiling with a systematic comparison of multiple health risk behaviors, and research on this topic among engineering students remains particularly scarce.

In summary, the present study targeted engineering college students and, guided by the SDT framework, employed LPA to identify distinct BPN profiles and compare students across profiles on multiple health risk behaviors (unhealthy weight control, traffic risk behavior, bullying victimization, loneliness, suicide risk, smoking, and internet addiction). The following hypotheses were tested: (1) BPN satisfaction among engineering college students is heterogeneous, and distinct latent profiles can be identified; (2) Students in different profiles differ significantly in health risk behaviors; (3) Students in the low-need-satisfaction profile exhibit more health risk behaviors.

## 2 Methods

### 2.1 Participants

Using convenience sampling, undergraduate engineering students at a university in Shandong Province, China, were surveyed between May and June 2021. Inclusion criteria were: (1) full-time undergraduate enrollment in an engineering program; (2) provision of informed consent. Exclusion criteria were: (1) incomplete questionnaire responses; (2) evidence of patterned responding. A total of 260 electronic questionnaires were distributed, yielding 251 valid responses (response rate: 96.5%). This study was approved by the Medical Ethics Committee of the School of Basic Medical Sciences, Shandong University (ECSBMSSDU2021-1-115).

### 2.2 Measures

#### 2.2.1 The general scale of satisfaction with basic needs

BPN satisfaction was assessed using the General Scale of Satisfaction with Basic Needs (BNSG-S), which measures three dimensions: autonomy, competence, and relatedness (19). The autonomy subscale measures individuals’ sense of volition and choice in activities and decisions; the competence subscale captures perceived ability and efficacy; and the relatedness subscale assesses feelings of connectedness and belonging with others. Higher scores on each dimension indicate greater need satisfaction. The Chinese version of the BNSG-S has been validated in college samples in mainland China (20). In the present study, Cronbach’s *α* coefficients for the three subscales were 0.862, 0.835, and 0.860, respectively.

#### 2.2.2 Health risk behavior indicators

Seven health risk behavior indicators were assessed, drawing on measurement approaches from the Global School-based Student Health Survey (GSHS) and the Youth Risk Behavior Surveillance System (YRBSS) (21): (1) *Unhealthy weight control*: Participants reported whether, in the past 30 days, they had intentionally vomited, fasted for 24 h or longer, or taken diet pills without medical supervision for the purpose of losing or controlling weight. Responses were summed to create a composite score, with higher scores indicating more unhealthy weight control behaviors. (2) *Traffic risk behavior*: Participants indicated how frequently they jaywalked (i.e., crossed the street without using crosswalks, overpasses, or underpasses) in the past 30 days, rated from 1 (*never*) to 5 (*always*). (3) *Bullying victimization*: Participants reported the frequency of being subjected to bullying behaviors (e.g., being ridiculed, having property extorted) in the past 30 days, with higher scores indicating more frequent victimization. (4) *Loneliness*: Participants rated the frequency of feeling lonely in the past 12 months on a scale from 1 (*never*) to 5 (*always*), with higher scores reflecting greater loneliness. (5) *Suicide risk*: A composite index combining suicidal ideation and suicide planning, scored from 0 to 3, with higher scores indicating greater suicide risk. (6) *Smoking frequency*: Participants reported the frequency of smoking in the past 30 days on a 7-point scale, with higher scores indicating more frequent smoking. (7) *Internet addiction*: Assessed with a 9-item scale developed by the research team consistent with widely used behavioral addiction frameworks (e.g., salience, withdrawal, tolerance, loss of control, conflict, and escape/mood modification). To ensure content validity, the item pool was reviewed and refined by a panel of psychology experts. A sample item is: “Even when not online, thoughts related to the internet keep coming to mind.” Items were scored 1 (*no*) or 2 (*yes*) and summed to yield a total score, with higher scores indicating greater internet addiction severity. Regarding psychometric properties: single-item indicators (traffic risk behavior, loneliness, and smoking frequency) and behavioral composite indices (unhealthy weight control and suicide risk) were drawn from the GSHS or YRBSS, instruments with established content and face validity across multinational adolescent samples; internal consistency is not applicable to single-item or formative composite measures. For the multi-item bullying victimization scale, Cronbach’s *α* = 0.958. For the internet addiction scale, Cronbach’s *α* = 0.840, indicating good internal consistency.

### 2.3 Data analysis

All analyses were conducted using R version 4.4.1. First, descriptive statistics and correlation analyses were performed for the three BPN dimensions. Second, LPA was conducted using the *tidyLPA* package, with autonomy, competence, and relatedness scores entered as indicator variables. Models specifying one to six profiles (equal variances, zero covariances) were estimated. The optimal number of profiles was determined based on the following criteria: (1) lower AIC and BIC values indicate better fit; (2) entropy values closer to 1.0 are preferred, with values > 0.80 indicating good classification accuracy; (3) the smallest class proportion should exceed 5% (22). Following profile identification, MANOVA was used to test overall between-profile differences across the seven risk behaviors, with Pillai’s Trace and Wilks’ Lambda reported. Where significant overall effects were found, one-way ANOVAs were conducted, with *η*^2^ reported as the effect size measure. Given the skewed distributions of several variables, Kruskal–Wallis nonparametric tests were also performed for verification. *Post hoc* pairwise comparisons were conducted using Dunn’s test with Holm correction. For the suicide risk variable, the IQR method was inapplicable due to a severe floor effect (*Q*1 = *Q*3 = 0); extreme values were therefore identified using a *Z*-score criterion (|*Z*| > 3), and a sensitivity analysis was conducted by re-running nonparametric tests after excluding identified extreme cases.

## 3 Results

### 3.1 Sample characteristics

A total of 251 engineering undergraduates participated. Demographic characteristics are presented in Table 1.

**Table 1:** Demographic characteristics of the sample (*N* = 251).

Variable	Category	*n* (%)
Gender	Male	229 (91.2%)
	Female	22 (8.8%)
Age (*M* ± *SD*)		18.96 ± 0.94
Year of study	Freshman	213 (84.9%)
	Sophomore	25 (10.0%)
	Junior	11 (4.4%)
	Senior	2 (0.8%)
Residence	Urban	184 (73.3%)
	Rural	67 (26.7%)
Single-parent family	Yes	19 (7.6%)
	No	232 (92.4%)

### 3.2 Descriptive statistics

Descriptive statistics for the three BPN dimensions and seven health risk behaviors are presented in Table 2. Moderate-to-high positive correlations were observed among the three BPN dimensions (autonomy–competence: *r* = 0.729; autonomy–relatedness: *r* = 0.703; competence–relatedness: *r* = 0.651; all *p*s < 0.001; Table 3).

**Table 2:** Descriptive statistics for basic psychological needs and health risk behaviors (*N* = 251).

Variable	*M*	*SD*	Min	Max
Autonomy	30.30	4.69	7	48
Competence	25.60	4.45	12	42
Relatedness	37.10	6.93	14	56
Unhealthy weight control	0.13	0.55	0	3
Traffic risk behavior	1.52	0.79	1	5
Bullying victimization	1.20	3.09	0	14
Loneliness	2.18	1.09	1	5
Suicide risk	0.15	0.55	0	3
Smoking frequency	1.49	1.47	1	7
Internet addiction	16.30	2.25	9	18

**Table 3:** Correlations among basic psychological need dimensions.

BPN dimension	Autonomy	Competence	Relatedness
Autonomy	1.000	0.729	0.703
Competence	0.729	1.000	0.651
Relatedness	0.703	0.651	1.000

### 3.3 Latent profile analysis

#### 3.3.1 Model fit comparisons

**Figure 1:**
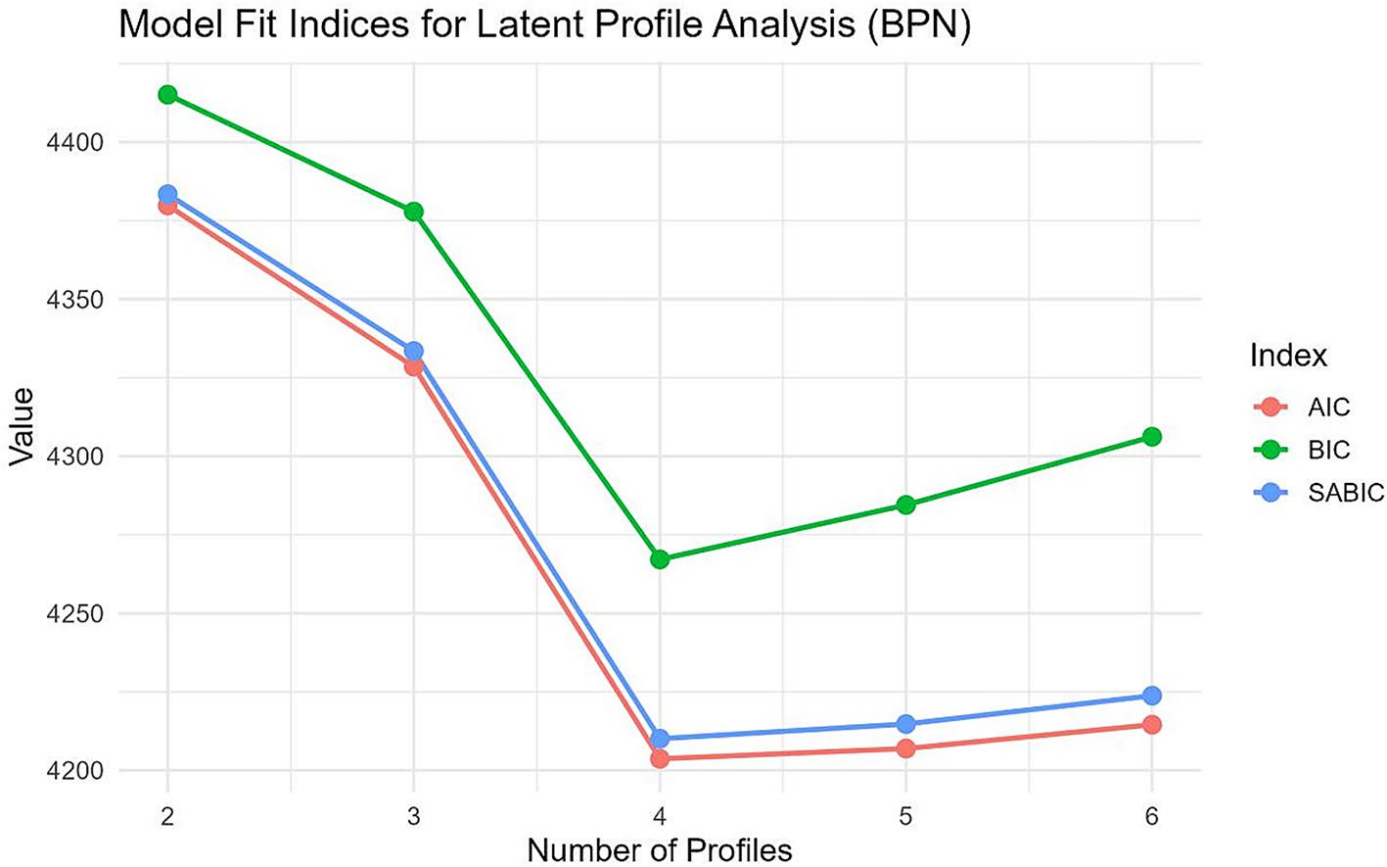
Model fit indices (AIC, BIC, SABIC) for 2- to 6-profile solutions.

LPA models specifying one to six profiles were estimated. Model fit indices are presented in Table 4; Figure 1. AIC and BIC values decreased progressively with increasing profile numbers. The three-profile model showed a substantial decline in BIC (4,378) relative to the two-profile model (4,415), with an entropy of 0.893—exceeding the 0.80 threshold for good classification—and a smallest class proportion of 5.6%, above the recommended 5% minimum. Although the four-profile model yielded a lower BIC (4,267), its smallest class comprised only 4.8% of the sample, falling below the 5% threshold. Moreover, entropy dropped sharply to 0.714 in the five-profile model, suggesting classification instability. Considering model fit, classification accuracy, and interpretability, the three-profile solution was selected as the final model. In supplementary analyses, the unequal-variance model (Model 2) failed to converge, further supporting the selection of the equal-variance three-profile model.

**Table 4:** Comparison of LPA model fit indices.

Profiles	AIC	BIC	SABIC	Entropy	Smallest class *n* (%)
1	4,642	4,664	4,645	1.000	251 (100%)
2	4,380	4,415	4,383	0.906	48 (19.1%)
**3**	**4,329**	**4,378**	**4,333**	**0.893**	**14 (5.6%)**
4	4,204	4,267	4,210	0.940	12 (4.8%)
5	4,207	4,284	4,215	0.714	12 (4.8%)
6	4,215	4,306	4,224	0.611	11 (4.4%)

Bold row indicates the selected model.

#### 3.3.2 Profile characteristics

**Figure 2:**
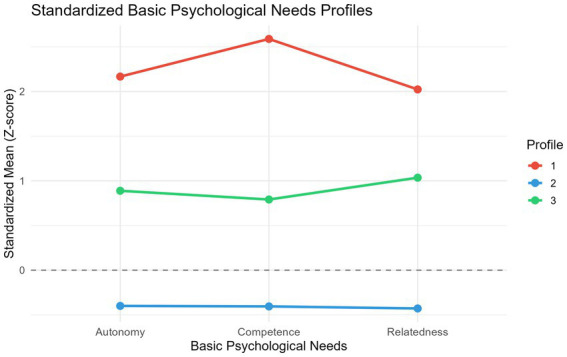
Standardized basic psychological needs scores across three latent profiles.

Average posterior probabilities for the three profiles were 0.944, 0.976, and 0.883, all exceeding the 0.70 acceptability threshold, indicating adequate classification precision. BPN scores across profiles are presented in Table 5; Figure 2. Based on the patterns of BPN scores, the three profiles were labeled as follows: *Profile 1: High-need-satisfaction (n = 14, 5.6%)*. Students in this profile scored substantially above the overall mean on all three dimensions—autonomy (*M* = 40.4), competence (*M* = 37.1), and relatedness (*M* = 51.1)—representing a group whose basic psychological needs were comprehensively and well satisfied. *Profile 2: Low-need-satisfaction (n = 187, 74.5%).* This was the largest profile. Students scored below the overall mean on all three dimensions—autonomy (*M* = 28.4), competence (*M* = 23.8), and relatedness (*M* = 34.2)—representing a group with relatively low BPN satisfaction. *Profile 3: Moderate-need-satisfaction (n = 50, 19.9%)*. Students in this profile scored at moderately high levels, falling between the high- and low-satisfaction profiles—autonomy (*M* = 34.4), competence (*M* = 29.1), and relatedness (*M* = 44.3)—representing a group with moderate BPN satisfaction.

**Table 5:** Basic psychological need characteristics by profile.

Profile	*n* (%)	Autonomy *M* (*SD*)	Competence *M* (*SD*)	Relatedness *M* (*SD*)
High-need-satisfaction	14 (5.6%)	40.40 (3.82)	37.10 (2.71)	51.10 (4.69)
Low-need-satisfaction	187 (74.5%)	28.40 (3.16)	23.80 (2.70)	34.20 (4.51)
Moderate-need-satisfaction	50 (19.9%)	34.40 (3.09)	29.10 (2.90)	44.30 (4.40)

### 3.4 Between-profile differences in health risk behaviors

#### 3.4.1 Overall differences

MANOVA results indicated a significant overall difference in the seven health risk behaviors across the three profiles (Pillai’s Trace = 0.103, *F*(14, 486) = 1.88, *p* = 0.026; Wilks’ Lambda = 0.899, *F*(14, 484) = 1.89, *p* = 0.025), indicating that students with different BPN satisfaction patterns differed significantly in their overall patterns of health risk behaviors.

#### 3.4.2 Differences in individual risk behaviors

**Figure 3:**
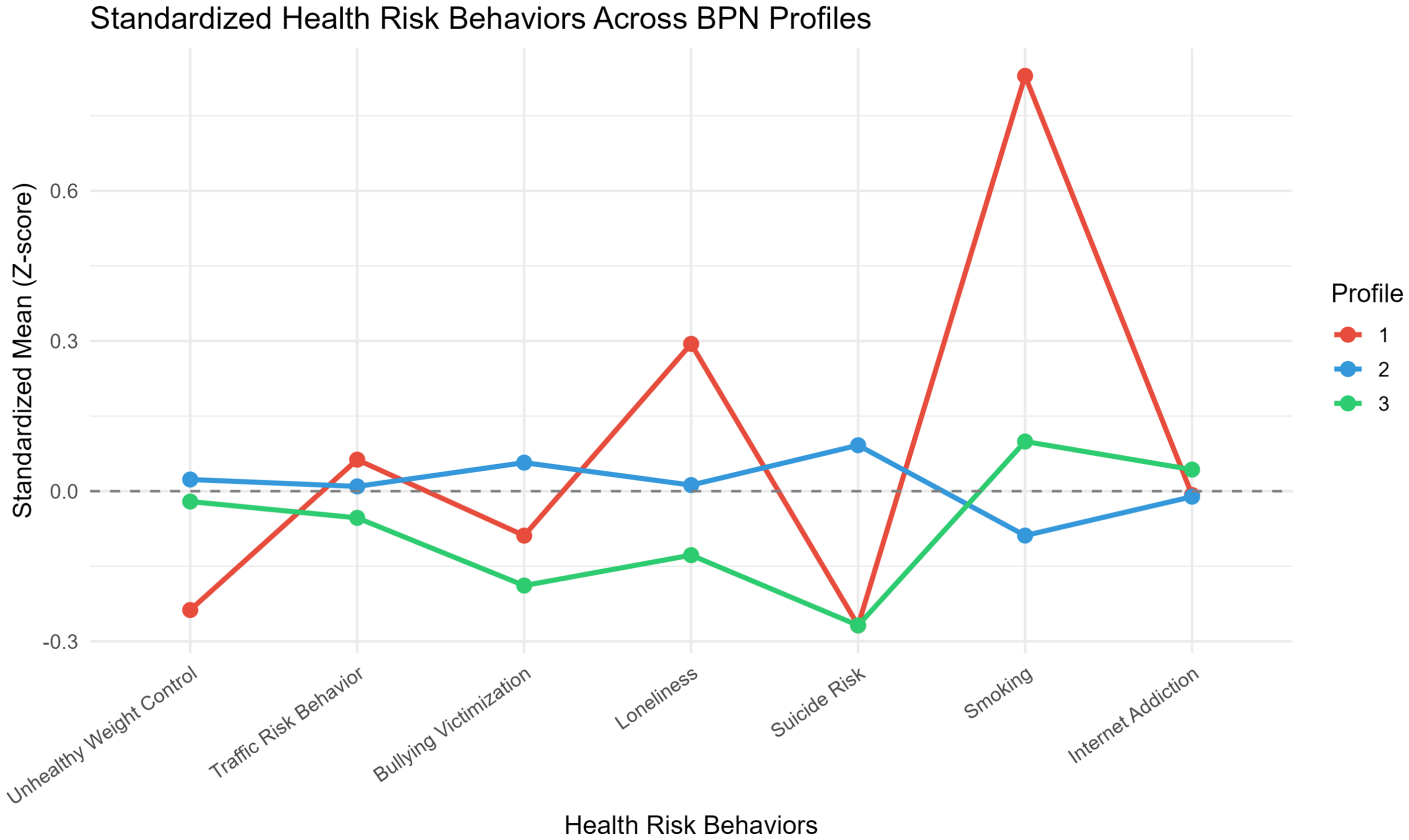
Standardized health risk behavior scores across three latent profiles.

Health risk behavior scores by profile are presented in Table 6; Figure 3; results of one-way ANOVAs and Kruskal–Wallis tests are shown in Table 7. Among the seven risk behaviors, suicide risk [*F*(2, 248) = 3.14, *p* = 0.045, *η*^2^ = 0.025] and smoking frequency [*F*(2, 248) = 6.03, *p* = 0.003, *η*^2^ = 0.046] showed significant between-profile differences, both with small effect sizes. The remaining five risk behaviors did not differ significantly across profiles (*p*s > 0.05). Kruskal–Wallis nonparametric tests produced consistent results, with significant differences for suicide risk (*χ*^2^ = 7.80, *p* = 0.020) and smoking frequency (*χ*^2^ = 8.32, *p* = 0.016) and nonsignificant results for all other variables.

**Table 6:** Health risk behavior scores by profile.

Risk behavior	High-need-satisfaction *M* (*SD*)	Low-need-satisfaction *M* (*SD*)	Moderate-need-satisfaction *M* (*SD*)
Unhealthy weight control	0.00 (0.00)	0.14 (0.58)	0.12 (0.52)
Traffic risk behavior	1.57 (1.16)	1.53 (0.78)	1.48 (0.71)
Bullying victimization	0.93 (1.94)	1.38 (3.35)	0.62 (2.17)
Loneliness	2.50 (1.22)	2.19 (1.10)	2.04 (0.99)
Suicide risk	0.00 (0.00)	0.20 (0.63)	0.00 (0.00)
Smoking frequency	2.71 (2.58)	1.36 (1.23)	1.64 (1.72)
Internet addiction	16.30 (2.58)	16.30 (2.29)	16.40 (2.03)

**Table 7:** Between-profile comparisons of health risk behaviors.

Risk behavior	ANOVA *F*	*p*	*η* ^2^	KW *χ*^2^	KW *p*
Unhealthy weight control	0.454	0.636	0.004	1.066	0.587
Traffic risk behavior	0.106	0.899	0.001	0.466	0.792
Bullying victimization	1.250	0.288	0.010	2.536	0.282
Loneliness	1.029	0.359	0.008	1.504	0.471
**Suicide risk**	**3.142**	0.045*	**0.025**	**7.796**	0.020*
**Smoking frequency**	**6.034**	0.003**	**0.046**	**8.319**	0.016*
Internet addiction	0.058	0.944	0.001	0.031	0.985

*p < 0.05, **p < 0.01. KW = Kruskal–Wallis test. Bold rows indicate significant variables.

#### 3.4.3 *Post hoc* comparisons

**Figure 4:**
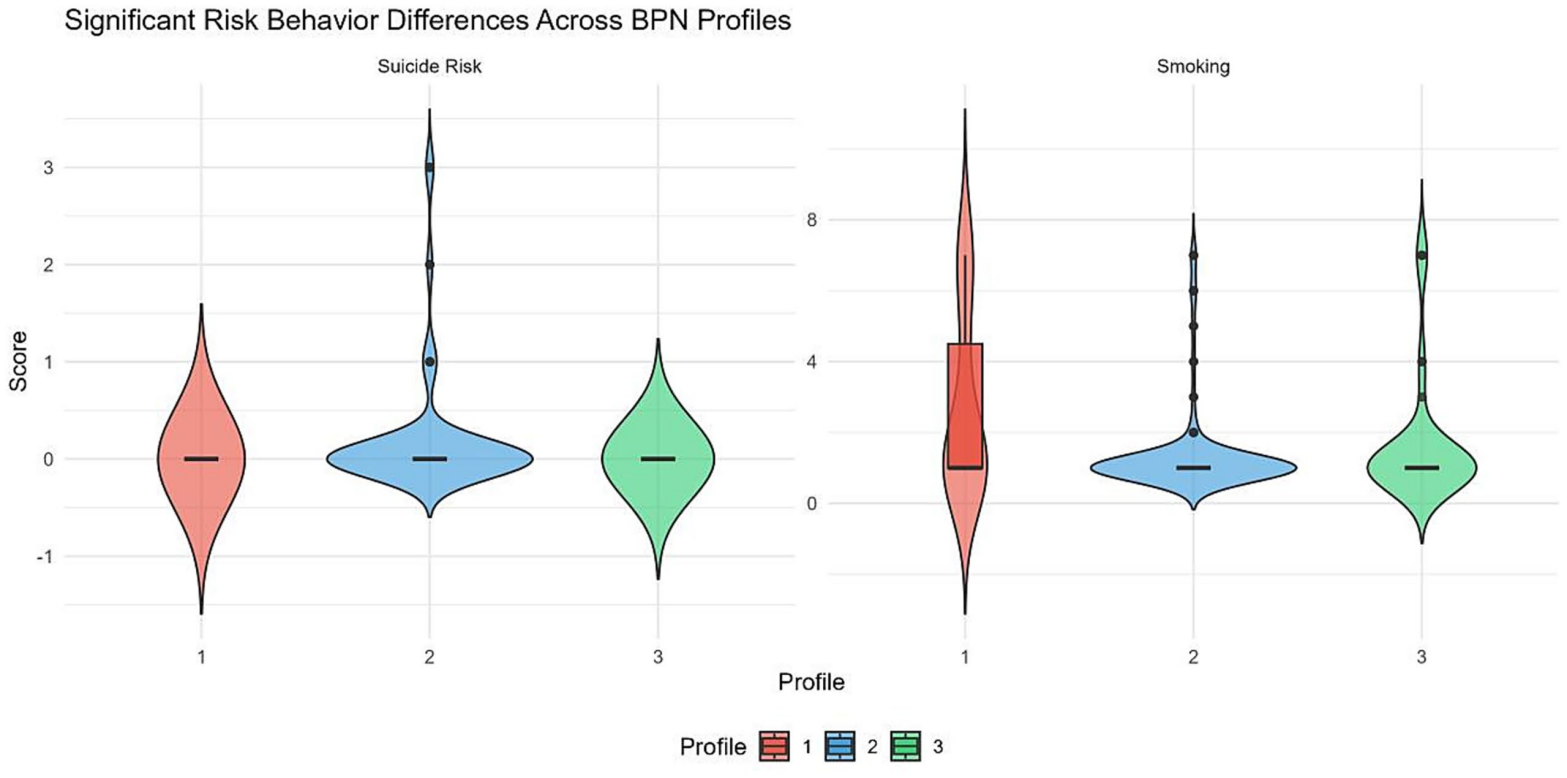
Distribution of suicide risk and smoking frequency across profiles (violin plot).

Because both the high-need-satisfaction and moderate-need-satisfaction profiles had zero-variance suicide risk scores (*SD* = 0), the variance assumption required for the Games–Howell test was not met. Therefore, Dunn’s test with Holm correction was used for *post hoc* comparisons of suicide risk, while both procedures were reported for smoking frequency. Results are presented in Table 8; Figure 4. Regarding suicide risk, the low-need-satisfaction profile scored significantly higher than the moderate-need-satisfaction profile (*p* = 0.033), whereas both the high-need-satisfaction and moderate-need-satisfaction profiles reported no suicide risk (*M* = 0). A sensitivity analysis excluding 10 extreme cases on suicide risk (|*Z*| > 3; *N* = 241) yielded a non-significant result [*χ*^2^(2) = 4.15, *p* = 0.126], indicating that the significant profile difference was sensitive to the presence of these extreme values. As these cases represent students with genuine suicidal ideation or planning, their removal does not negate the clinical relevance of the pattern, but the finding should be interpreted with caution. Regarding smoking, the high-need-satisfaction profile smoked significantly more frequently than the low-need-satisfaction profile (*p* = 0.013).

**Table 8:** *Post hoc* comparisons for significant variables (Dunn’s test, Holm correction).

Variable	Comparison	*Z*	*p*_adj	Significance
Suicide risk	High vs. low	1.46	0.289	—
	High vs. moderate	0.00	1.000	—
	Low vs. moderate	−2.54	0.033	*
Smoking frequency	High vs. low	−2.86	0.013	*
	High vs. moderate	−2.23	0.051	—
	Low vs. moderate	0.74	0.462	—

^*^p < 0.05.

## 4 Discussion

### 4.1 Latent profiles of basic psychological needs among engineering students

Using LPA, this study was the first to identify three distinct BPN satisfaction patterns among engineering college students: high-need-satisfaction (5.6%), low-need-satisfaction (74.5%), and moderate-need-satisfaction (19.9%). This finding supports Hypothesis 1, demonstrating significant group heterogeneity in BPN satisfaction among engineering students. It is consistent with prior person-centered research on BPN; for example, Gu et al. (8) similarly identified multiple need satisfaction profiles among adolescents and college students.

Notably, the low-need-satisfaction profile accounted for 74.5% of the sample, indicating that the vast majority of engineering students did not have their autonomy, competence, and relatedness needs adequately met. This finding may reflect characteristics inherent to engineering education—heavy academic demands, highly structured curricula, and limited autonomy in course selection—which may, to some extent, constrain the satisfaction of autonomy and competence needs (23). Additionally, the low proportion of female students in engineering programs creates a relatively homogeneous social ecology that may hinder relatedness satisfaction. These findings suggest that universities should attend to the BPN satisfaction of engineering students and seek to promote need fulfillment by enhancing curricular flexibility, providing opportunities for competence development, and fostering supportive social environments. The three profiles displayed a consistent high–moderate–low gradient across all three dimensions, suggesting that the three needs tend to covary at the individual level. This is consistent with a core assumption of SDT—that the three basic psychological needs are mutually reinforcing, such that satisfaction of one need facilitates satisfaction of the others (24). The moderate-to-high positive correlations among the three dimensions (*r* = 0.651–0.729) observed in this study further support this view.

### 4.2 BPN profiles and suicide risk

The present study found significant between-profile differences in suicide risk (*p* = 0.045), partially supporting Hypotheses 2 and 3. Specifically, students in the low-need-satisfaction profile exhibited significantly higher suicide risk than those in the moderate-need-satisfaction profile, whereas neither the high-need-satisfaction nor the moderate-need-satisfaction profile reported any suicide risk. This result aligns with SDT’s prediction that need frustration is associated with psychological distress and maladaptation (25). From a theoretical perspective, the interpersonal theory of suicide posits that thwarted belongingness and perceived burdensomeness are proximal risk factors for suicidal ideation (26). In this study, students in the low-need-satisfaction profile scored low on all three needs; deficits in relatedness may be associated with thwarted belongingness, while inadequate competence satisfaction may be related to perceived burdensomeness, both of which may in turn be linked to elevated suicide risk. Tucker and Wingate (27) demonstrated that BPN frustration predicted subsequent suicidal ideation, and this association remained significant even after controlling for depressive symptoms. The present findings provide further support for this theoretical account from a person-centered perspective. It is worth noting that even within the low-need-satisfaction profile, mean suicide risk was relatively low (*M* = 0.20), and the overall incidence of suicide risk in the sample was modest, potentially limiting statistical power. Nevertheless, given the clinical significance of suicide risk, even small effect sizes carry important practical implications. It is also important to note that the effect size for this difference was small (*η*^2^ = 0.025), and a sensitivity analysis excluding extreme outliers rendered this difference non-significant [*χ*^2^(2) = 4.15, *p* = 0.126]. This indicates that the significant profile difference in suicide risk is sensitive to the inclusion of extreme values. These findings should therefore be interpreted with considerable caution and require replication in larger, more representative samples before firm conclusions can be drawn.

### 4.3 BPN profiles and smoking behavior

Smoking behavior differed significantly across profiles (*p* = 0.003, *η*^2^ = 0.046), representing the largest effect size observed in this study, but this effect size remains small by conventional standards, and the result should be interpreted accordingly. However, *post hoc* comparisons revealed that students in the high-need-satisfaction profile smoked more frequently (*M* = 2.71) than those in the low-need-satisfaction profile (*M* = 1.36)—a finding that appears inconsistent with SDT predictions. Several considerations may help explain this pattern. First, and most critically, the high-need-satisfaction profile comprised only 14 students. The group mean (*M* = 2.71) is highly susceptible to distortion by even one or two outlying individuals, and may not be a statistically stable estimate of the true mean for this latent profile. The large standard deviation (*SD* = 2.58) confirms substantial within-group heterogeneity and raises questions about the reliability of this group estimate. As a consequence, the significant smoking difference between profiles should be interpreted with great caution. This finding cannot be considered robust until replicated in samples with a substantially larger high-need-satisfaction subgroup. Second, prior research has suggested that autonomy satisfaction within SDT may drive individuals to seek novel and stimulating experiences, a tendency potentially linked to sensation seeking (28). Among college students, smoking is sometimes associated with social activities, and individuals with high relatedness satisfaction may encounter smoking behaviors more frequently in social contexts. Third, smoking behavior among Chinese college students is also influenced by environmental factors such as psychological distress, academic burnout, and peer influence (29), which may operate independently of BPN.

### 4.4 Profile differences in other health risk behaviors

From a theoretical standpoint, the selective emergence of significant differences in suicide risk and smoking, but not the other five behaviors, is consistent with SDT’s account of need frustration. Suicide risk and smoking represent outcomes that are proximally driven by internal psychological states: chronic need deprivation is theorized to generate psychological pain and dysregulated coping (5), which may more directly manifest as suicidal ideation (25, 26) or the use of smoking as a mood-regulatory strategy (28). By contrast, behaviors such as bullying victimization, internet addiction, traffic risk, loneliness, and unhealthy weight control are more strongly shaped by external contextual factors, including peer dynamics, environmental affordances, cultural norms, and social learning, which operate with greater independence from need satisfaction levels (11, 12). This may explain why BPN profiles differentiated students on the former behaviors but not the latter.

Unhealthy weight control, traffic risk behavior, bullying victimization, loneliness, and internet addiction did not differ significantly across profiles. These nonsignificant findings can be understood from several perspectives. With respect to sample characteristics, several variables exhibited pronounced floor effects—for instance, the vast majority of students scored zero on unhealthy weight control—and such extreme distributional skewness substantially limits discriminative validity. Because the sample was predominantly male, behaviors more commonly reported among women (e.g., unhealthy weight control) had very low prevalence, potentially masking underlying associations. Furthermore, the small size of the high-need-satisfaction profile (*n* = 14) inherently limited the statistical power required to detect small effect sizes. Nonetheless, the significant overall MANOVA result (*p* = 0.026) indicates that BPN profiles were meaningfully related to the composite pattern of health risk behaviors, even though individual variable effects did not all reach significance.

### 4.5 Theoretical and practical implications

This study makes both theoretical and practical contributions. At the theoretical level, it extends the application of SDT to health risk behavior research by being the first to use a person-centered approach to demonstrate heterogeneity in BPN satisfaction among engineering college students and its association with risk behaviors. These findings enrich our understanding of the “need satisfaction–behavioral adaptation” link by revealing how individuals with different need satisfaction patterns differ in specific risk behaviors, particularly suicide risk. At the practical level, several implications for university mental health services can be drawn. First, based on the present exploratory and cross-sectional findings, low BPN satisfaction may be tentatively considered a potential correlate of suicide risk. However, given the small effect size (*η*^2^ = 0.025) and the sensitivity of the data to extreme values, it would be premature to recommend BPN assessment as a standalone early warning tool. Rather, these preliminary results suggest that future longitudinal and intervention studies are needed to investigate whether systematically supporting students’ psychological needs is associated with reductions in suicide-related outcomes. If replicated over time, such findings could eventually inform broader university mental health screening practices. Second, intervention strategies should focus on need satisfaction—specifically, by providing autonomy support, offering opportunities for competence development, and creating socially inclusive environments that foster a sense of belonging (30). Given the high prevalence of low BPN satisfaction (74.5%) in this sample, system-level environmental improvements at the departmental or institutional level may be more effective and equitable than individually targeted interventions. In this regard, structured physical education courses, particularly team-based activities such as basketball, may serve as a practically accessible avenue for need-supportive intervention among engineering students, as they offer integrated opportunities to foster relatedness through cooperative play, build competence through skill acquisition, and reinforce autonomy through self-directed engagement. Future studies should examine empirically whether such programmes are associated with improved BPN satisfaction and reductions in need-frustration-driven outcomes.

### 4.6 Limitations and future directions

This study has several limitations. First, the sample was drawn from a single university’s engineering program and was predominantly male freshmen, which limits generalizability. However, the sample composition closely mirrors the actual gender distribution in Chinese engineering programs, lending a degree of ecological validity. Future research should recruit larger, multi-institutional, and multi-disciplinary samples to enhance external validity. Second, the cross-sectional design precludes causal inferences. Longitudinal or experimental studies are needed to establish the directionality of the relationship between BPN satisfaction and health risk behaviors. Third, the smallest profile in the three-class solution contained only 14 participants (5.6%). Although this marginally exceeds the conventional minimum threshold for LPA (>5%), such a small subgroup raises substantial concerns about statistical stability. Specifically, the group-level mean for smoking in this profile (*M* = 2.71, *SD* = 2.58) is vulnerable to undue influence from individual extreme scorers, limiting the generalizability of the between-profile smoking comparison. Future studies with larger samples should replicate this finding before drawing any substantive conclusions about the smoking behavior of individuals with high BPN satisfaction. Fourth, several measurement constraints may have influenced the results. Three health risk behavior indicators (traffic risk behavior, loneliness, and smoking frequency) were assessed with single items. Single-item measures have inherently limited reliability and cannot capture the full construct breadth, which may have attenuated the observed associations between BPN profiles and these behaviors, contributing to the non-significant findings for these variables. Additionally, pronounced floor effects were observed for certain variables, most notably unhealthy weight control and suicide risk, where the majority of participants scored zero. Floor effects severely restrict variance and reduce the discriminative capacity of these measures, making it difficult to detect meaningful between-profile differences even if they exist. Future studies should consider using validated multi-item scales for all health risk behavior indicators and should target populations with higher base rates of these behaviors—such as clinical samples, high school students, or vocational college students—where floor effects are less likely to constrain the analyses. Fifth, demographic variables such as gender and year of study were not included as covariates. Given the high homogeneity of the sample (91.2% male, 84.9% freshmen), between-group variability in demographic variables was limited, and including covariates could have introduced estimation instability. Future studies with more balanced samples in terms of gender and academic year may adopt a three-step approach to further examine the effects of demographic factors on profile membership and risk behavior outcomes.

## 5 Conclusion

Grounded in self-determination theory, this study employed latent profile analysis and identified three distinct BPN satisfaction patterns among engineering college students: high-need-satisfaction, low-need-satisfaction, and moderate-need-satisfaction. Significant overall differences in health risk behaviors were found across profiles, with students in the low-need-satisfaction profile exhibiting significantly higher suicide risk than those in the moderate-need-satisfaction profile. These results indicate that BPN satisfaction is an important factor associated with health risk behaviors among college students, which is consistent with a core proposition of SDT. University mental health services should attend to students with low levels of BPN satisfaction and promoting need fulfillment may represent a promising avenue for supporting student psychological well-being. However, it is notable that only two of the seven health risk behaviors showed statistically significant between-profile differences, both with small effect sizes, and the finding for suicide risk was sensitive to the removal of extreme values. These results should therefore be interpreted cautiously as preliminary and exploratory.

## Data Availability

The raw data supporting the conclusions of this article will be made available by the authors, without undue reservation.
